# Practical surrogate marker of pulmonary dysanapsis by simple spirometry: an observational case–control study in primary care

**DOI:** 10.1186/s12875-015-0255-4

**Published:** 2015-03-26

**Authors:** Satomi Shiota, Masako Ichikawa, Kazuhiro Suzuki, Yoshinosuke Fukuchi, Kazuhisa Takahashi

**Affiliations:** Department of Respiratory Medicine, Juntendo University Graduate School of Medicine, 2-1-1 Hongo, 113-8421 Bunkyo-ku, Tokyo Japan; Department of Radiology, Juntendo University Graduate School of Medicine, 2-1-1 Hongo, 113-8421 Bunkyo-ku, Tokyo Japan

**Keywords:** Dysanapsis, Airflow limitation, Spirometry, Surrogate marker

## Abstract

**Background:**

We see patients who present with spirometry airflow limitation despite their forced expiratory volume in one second (FEV_1_) as well as forced vital capacity (FVC) to be supernormal (FEV_1_/FVC < 70%, both the %FEV_1_ and the %FVC ≧100%) in asymptomatic healthy non-smokers. Based on previous studies, we hypothesized these spirometry conditions (results measured with spirometry) could be suitably used as a practical surrogate marker of pulmonary dysanapsis: the condition of disproportionate but physiologically normal growth between airways and lung parenchyma.

**Methods:**

We compared the conventional surrogate marker of dysanapsis, maximum mid-expiratory flow to FVC (MMF/FVC), in SUBJECTS (FEV_1_/FVC < 70%, both the %FEV_1_ and the %FVC ≧100% in healthy non-smokers) (n = 25), in EMPHYSEMA (CT confirmed pulmonary emphysema, same spirometry results with SUBJECTS) (n = 55), and in CONTROLS (age- and height- matched, normal spirometry results) (n = 25). Next we added imaging analysis to evaluate the relationship between the cross sectional airway luminal area (X-Ai) and the lung volume results among the three groups.

**Results:**

The MMF/FVC was significantly lower in SUBJECTS and in EMPHYSEMA compared to CONTROLS. However, percent predicted peak expiratory flow (%PEFR) was significantly lower only in SUBJECTS and not in EMPHYSEMA compared to CONTROLS. The ratio of the X-Ai of the trachea and right apical bronchus to lung volume was significantly lower in SUBJECTS compared to CONTROLS.

**Conclusion:**

The simple spirometry conditions in SUBJECTS are highly suggestive of practical surrogate marker of pulmonary dysanapsis. Awareness of this concept would help to attenuate the risk of overdiagnosis of obstructive pulmonary disease.

## Background

A globally accepted definition of airflow limitation by spirometry is when the forced expiratory volume in one second divided by the forced vital capacity (FEV1/FVC) ratio is less than 70%. In daily clinical situations, including screening, we see patients who, from spirometry, present with airflow limitation despite being in the supernormal.

percent predicted of FEV1 as well as FVC in healthy non-smokers who have neither airway symptoms nor any previous diagnosis of pulmonary disease. Thus, it is difficult to correctly clarify their pathophysiological states and may sometimes lead to overdiagnosis of obstructive pulmonary disease. People with large lungs do not necessarily have larger airways than people with small lungs. The concept of pulmonary dysanapsis was originally described as disproportionate but physiologically normal growth between airways and lung parenchyma [1]. Variability between individual’s lung volume and airway size were accessed indirectly or directly [2,3]. Later, the ratio of maximal mid-expiratory flow to FVC (MMF/FVC) has been accepted as a surrogate marker of dysanapsis [4], based on the concept of this ratio’s close relationship with the previously reported ratio by Mead [2]. MMF/FVC can be obtained by conventional spirometry, however this ratio has not been established as a cut-off value to define dysanapsis. Furthermore, this ratio is usually not present and not calculated on conventional spirometry reports, making it difficult to diagnose dysanapsis from these reports alone. We focused on subjects that were healthy non-smokers, but who presented with airflow limitation on spirometry whilst having ‘supernormal’ percent predicted FEV1 as well as FVC (FEV1/FVC < 70%, % FEV1 and FVC≧100%). We hypothesized that this simple spirometry parameter indicated and could be used as another practical surrogate marker of pulmonary dysanapsis. We combined the evaluation of spirometry results with the evaluation from imaging analysis on the above-mentioned subjects. We also compared them to subjects with emphysema that had similar spirometry findings and also to age- and height- matched controls that had normal spirometry findings.

## Methods & study subjects

We retrospectively reviewed male subjects, from April 2008 to April 2013, whose data were available for both spirometry and for chest computed tomography (CT) in our institute. We identified 25 asymptomatic healthy non-smokers with normal CT image (SUBJECTS) and 55 CT-diagnosed pulmonary emphysema (EMPHYSEMA), both groups demonstrated airflow limitation but FEV1 and FVC were still within the supernormal percent predicted (i.e. FEV1/FVC < 70%, %FEV1 and %FVC≧100%). The diagnosis of pulmonary emphysema was based on the spirometric criteria of the Global Initiative for Chronic Obstructive Lung Disease (GOLD) guideline [5] and by CT findings confirmed subjectively by a respirologist and a radiologist. We also enrolled 25 asymptomatic healthy never-smokers who matched the age and height (CONTROLS) of those who underwent the annual health check. We only enrolled men considering gender differences of airways for a given lung volume, even when corrected for age and height [2]. We excluded patients who (i) did not complete both spirometry and CT tests, (ii) had incomplete medical records, (iii) had pulmonary disease including those with pulmonary nodules of more than 3 cm in diameter, and (iii) had image artifacts that could have potentially interfered with image analysis. This retrospective and observational study was approved by the ethics committee of our institution. Spirometry was conducted according to the American Thoracic Society (ATS) recommendations [6] and to a standard technique using computerized pneumotachograph based equipment (Minato Medical Science, Osaka, JAPAN). The reference values obtained from the Japanese population were utilized to calculate the % predicted values [7]. Spirometry was not specifically completed after a bronchodilator. Sequential CT scans were obtained using the 64-detectorrow CT scanner (Aquilion-64; Toshiba Medical, Tokyo, Japan) with a 2- to 10- mm slice thickness, scanning parameters of 120 kVp, auto exposure control (target SD 10), and a field of view of 32 cm. All of SUBJECTS, EMPHYSEMA and CONTROLS had undergone CT imaging to be evaluated the lung parenchyma. Among them, we applied CT imaging analysis on 10 SUBJECTS, 21 EMPHYSEMA and 16 CONTROLS whose CT conditions were available to apply imaging analysis software (Airway Inspector, Brigham and Women’s Hospital, Boston, MA) [8]. The CT images in this cohort study were reviewed by a respiratory specialist and by a radiologist. We measured the cross sectional airway luminal area (X-Ai) of the inner lumen and each wall area (WA) percent [100 × ((total area-lumen area)/total area)] at the 3 levels: (i) trachea above aortic arch (Tr), (ii) right apical segmental bronchus (rB1) and, (iii) right mediobasal segmental bronchus (rB7). These levels were chosen because they were located in a plane relatively perpendicular to the transverse plane of image acquisition. Due to the wide range of slice thickness in our clinically acquired CT data, we visually assessed the severity of emphysema on CT according to the modified Goddard scoring system [9], instead of assessing Hounsfield Unit threshold using imaging analysis software. Six images were analyzed in three slices in the lungs and an average score of all images was considered as a representative value of the severity of emphysema in each person. Each image was classified as normal (score 0), (5% affected (score 0.5), (25% affected (score 1), (50% affected (score 2), (75% affected (score 3) and .75% affected (score 4), giving a minimum score of 0 and maximum of 4.

Statistical analysis of variance and kruskal-wallis test were performed using SPSS Vers 20 (IBM, Chicago, IL). Data were expressed as mean ± standard deviation. For all statistical analysis, *p* value of less than .05 was considered to show a statistically significant difference.

### Ethics

This project is retrospective medical data base research and required to be notified to the research ethics committee. Ethics approval for the study was received from the Juntendo University School of Medicine Research Ethics Committee (No. 14–028).

## Results

**Figure 1:**
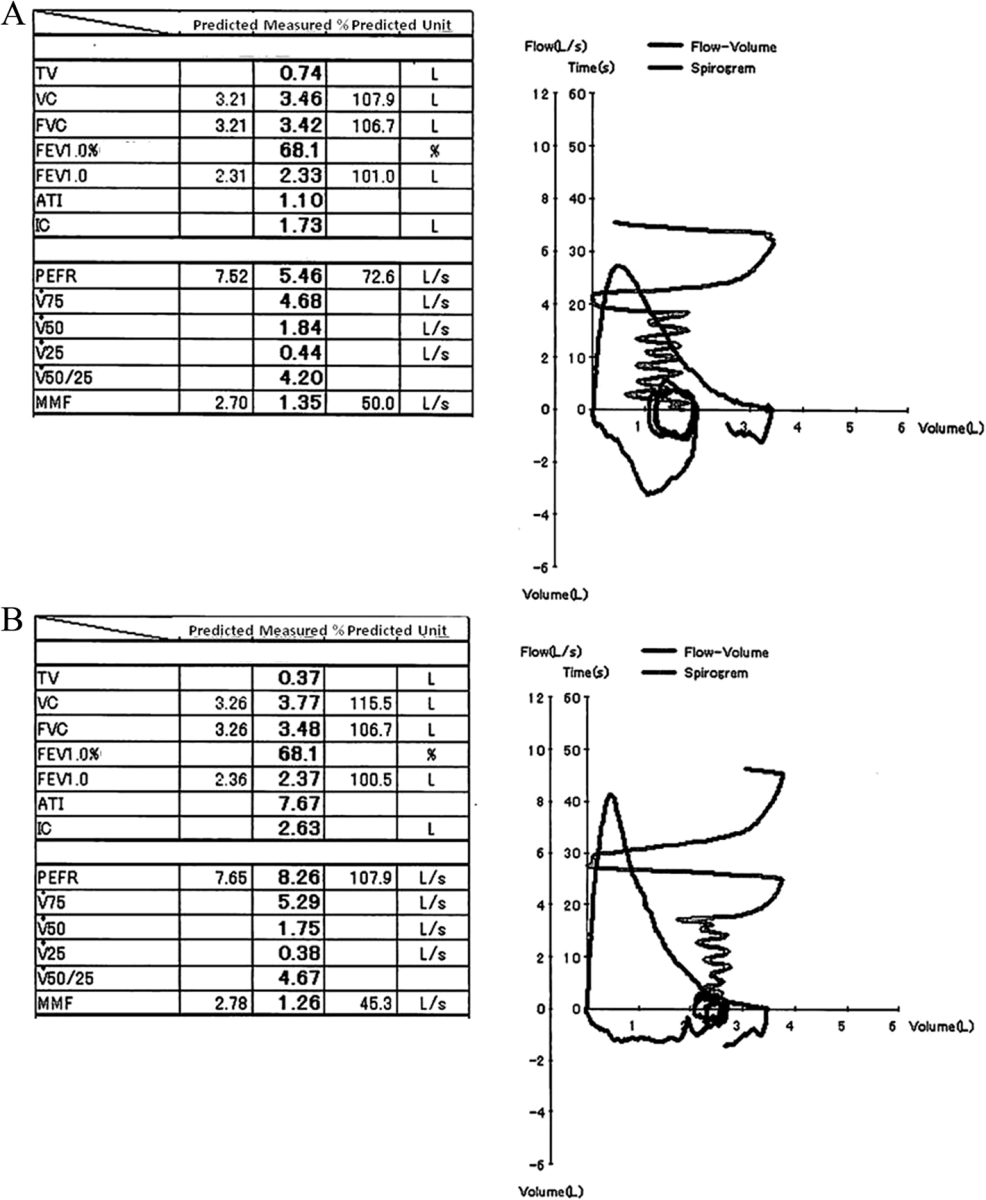
**Representative cases. A**. Representative spirometry results of SUBJECTS. (75y.o., 159.1 cm, BSA 1.60 mm^2^, non-smoker). **B**. Representative spirometry results of EMPHYSEMA. (74y.o., 160.7 cm, BSA 1.70 mm^2^, 78 packs per year tobacco history).

The age-matched representative spirometry results of SUBJECTS and EMPHYSEMA are shown in Figure 1A and B, respectively. Between SUBJECTS and EMPHYSEMA, their FEV1/FVC results were closely matched. Notably, on the other hand, %PEFR was clearly different between the two groups.

Descriptive characteristics of the entire study population, including age, height, body mass index (BMI) and spirometry results are provided in Table 1. Unsurprisingly, SUBJECTS and EMPHYSEMA showed higher lung volume and lower FEV1/FVC compared to CONTROLS, as these was the initial enrollment criteria in this study. MMF/FVC, which was the classical surrogate marker of dysanapsis, was significantly lower in SUBJECTS as well as EMPHYSEMA compared to CONTROLS. Notably, on the other hand, %PEFR which relatively represents central airway, was significantly lower only in SUBJECTS and not in EMPHYSEMA compared to CONTROLS as we presented in Figure 1. In addition, MMF which relatively represents small airway was higher in SUBJECTS compared to EMPHYSEMA. The mean modified Goddard score was 1.53 (1.00-2.67) in EMPHYSEMA (data not shown).

**Table 1:** **Patient’s characteristics and Spirometry results**

	**SUBJECTS (n = 25)**			**EMPHYSEMA (n = 55)**			**CONTROLS (n = 25)**		
Age, years	72.8	±	7.0^**†**^	68.2	±	7.4	71.0	±	7.7
Height, cm	166.0	±	7.8	168.2	±	5.9	165.8	±	5.9
BMI, kg/mm^2^	22.7	±	2.7**	22.2	±	2.6^**‡‡**^	26.0	±	3.8
BSA, mm^2^	1.7	±	0.2	1.7	±	0.1	1.8	±	0.2
**VC, %pred**	**120.4**	**±**	9.8**	**125.2**	**±**	**9.8** ^**‡‡**^	100.3	±	16.0
**FVC, %pred**	**114.7**	**±**	8.2**	**119.4**	**±**	**8.1** ^**‡‡**^	95.6	±	15.4
**FEV1**, %pred	106.1	±	7.1	105.4	±	5.8	101.5	±	13.8
**FEV1/FVC, %**	**67.2**	**±**	2.6**	**65.6**	**±**	**3.2** ^**‡‡**^	78.6	±	4.3
**PEFR, %pred**	**99.6**	**±**	13.8**	**104.9**	**±**	**10.1**	111.6	±	18.1
**MMF, %pred**	**48.6**	**±**	7.7** ^**††**^	**43.9**	**±**	**6.0** ^**‡‡**^	76.2	±	19.8
V50/V25	4.6	±	1.46	4.38	±	1.11	4.21	±	1.22
ATI	4.61	±	3.7	4.52	±	2.96	5.22	±	2.94
**MMF/FVC**	**0.37**	**±**	0.05** ^**††**^	**0.33**	**±**	**0.05** ^**‡‡**^	0.88	±	0.17

Values are mean ± SD. BMI, body mass index; BSA, body surface area; VC, vital

capacity; FVC, forced vital capacity; FEV1, forced expiratory volume in one second; PEFR, peak expiratory flow; MMF, maximal mid-expiratory flow; V50 and V25, Maximum expiratory flow rates at 50% and 25% of the FVC; ATI, air trapping index.

**Significantly different from CONTROLS (p < 0.01). ^**††**^Significantly different from EMPHYSEMA (p < 0.01). ^**†**^Significantly different from EMPHYSEMA (p < 0.05). ^**‡‡**^Significantly different from CONTROLS (p < 0.01).

**Figure 2:**
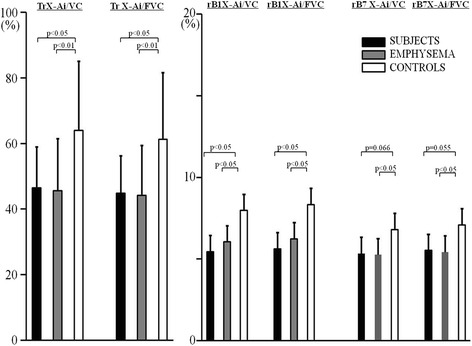
**The cross sectional airway luminal area (X-Ai) to lung volume at the level of Trachea, right B1 and right B7.** For definition of abbreviations *see* Table 1 and Table 3.

Descriptive characteristics of the available study population who underwent CT imaging analysis were provided in Table 2. The average time interval between spirometry and CT was 7.7 months. Similar results to those in Table 1 were found. Descriptive characteristics, focusing on the ratio of X-Ai to lung volume by CT imaging analysis, were provided in Table 3 and graphed in Figure 2. The ratio of the inner X-Ai to lung size at the level of Tr and rB1 were found to be significantly lower in both SUBJECTS and EMPHYSEMA, compared to CONTROLS. Even at the level of rB7, the ratio of the inner X-Ai to lung volume showed tendency to be significant lower in SUBJECTS compared to CONTROLS (Figure 2). WA % showed no significant difference among the three groups.

**Table 2:** **Descriptive characteristics of the available study population who underwent CT analysis**

**Group**	**SUBJECTS (n = 10)**			**EMPHYSEMA (n = 21)**			**CONTROLS (n = 16)**		
Age, years	72.6	±	7.2	67.1	±	6.0	69.8	±	8.7
Height, cm	168.4	±	10.5	168.2	±	5.8	167.1	±	6.4
BMI, kg/mm^2^	22.2	±	1.9*	22.0	±	3.2^**‡‡**^	26.0	±	3.4
BSA, mm^2^	1.7	±	0.2	1.7	±	0.1	1.8	±	0.2
**VC, %pred**	**118.9**	**±**	10.0**	**122.9**	**±**	**8.8** ^**‡‡**^	99.6	±	14.1
**FVC, %pred**	**114.7**	**±**	9.9*	**119.2**	**±**	**8.0** ^**††**^	95.2	±	13.0
**FEV1**, %pred	106.8	±	8.6	105.0	±	6.3	101.7	±	11.3
**FEV1/FVC, %**	**67.7**	**±**	2.1**	**65.8**	**±**	**3.1** ^**‡‡**^	79.6	±	4.2
**PEFR, %pred**	**94.4**	**±**	14.8**	104.8	±	10.0	114.9	±	18.4
**MMF, %pred**	**51.7**	**±**	8.1**	**44.2**	**±**	**5.5** ^**‡‡**^	80.2	±	19.9
V50/V25	4.5	±	1.9	4.6	±	1.3	4.0	±	1.2
ATI	3.5	±	3.8	3.0	±	1.9	4.2	±	2.4
**MMF/FVC**	**0.39**	**±**	0.05** ^**††**^	**0.34**	**±**	**0.04** ^**‡‡**^	0.75	±	0.17

Values are mean ± SD. For definition of abbreviations *see* Table 1.

**Significantly different from CONTROLS (p < 0.01).

* Significantly different from CONTROLS (p < 0.05).

^**††**^Significantly different from EMPHYSEMA (p < 0.01). ^**‡‡**^Significantly different from CONTROLS (p < 0.01).

**Table 3:** **Descriptive characteristics, focusing on the ratio of airway X-Ai to lung volume of the available study population who underwent CT analysis**

**Group**	**SUBJECTS (n = 10)**			**EMPHYSEMA (n = 21)**			**CONTROLS (n = 16)**		
Tr, WA, %	21.0	±	3.6	22.1	±	5.6	22.4	±	3.1
rB1 WA, %	52.1	±	5.1	49.1	±	5.0	49.6	±	4.4
rB7 WA, %	51.8	±	3.7	51.4	±	4.64	50.9	±	3.8
**TrX-Ai/VC**	**44.8**	**±**	11.5*	**44.2**	**±**	**15.3** ^**$$**^	61.3	±	20.3
**Tr X-Ai /FVC**	**46.5**	**±**	12.4*	**45.6**	**±**	**15.9** ^**$$**^	64.0	±	21.2
**rB1X-Ai /VC**	**5.45**	**±**	2.30*	**6.05**	**±**	**2.05** ^**$**^	8.0	±	2.3
**rB1X-Ai /FVC**	**5.62**	**±**	2.28*	**6.23**	**±**	**2.09** ^**$**^	8.3	±	2.5
**rB7 X-Ai /VC**	**5.34**	**±**	**1.16**	**5.26**	**±**	**1.51** ^**$**^	6.80	±	1.85
**rB7X-Ai /FVC**	**5.52**	**±**	**1.1 2**	**5.43**	**±**	**1.58** ^**$**^	7.09	±	1.92

Values are mean ± SD. Trachea; Tr, Wall area; WA, Right B1; rB1, Right B7; rB7, inner cross sectional luminal area at trachea; TrX-Ai, inner cross sectional luminal area at rB1; rB1 X-Ai, inner cross sectional luminal area at rB7; rB7 X-Ai.

* Significantly different from CONTROLS (p < 0.05).

^$^Significantly different from CONTROLS (p < 0.05).

^$$^Significantly different from CONTROLS (p < 0.01).

## Discussion

### Main findings

In the present clinical study, some novel observations could be made. Firstly, compared to CONTROLS, SUBJECTS showed significantly lower MMF/FVC ratio which has been previously reported as a surrogate marker of pulmonary dysanapsis [4]. Secondly, compared to CONTROLS, SUBJECTS also showed a significant lower ratio of airway size to lung volume at the level of Tr and rB1, which is the principle concept of ‘dysanapsis condition’. Thirdly, EMPHYSEMA, with the same spirometry conditions, also showed a significant lower MMF/FVC ratio and a lower ratio of airway size to lung volume at the level of Tr, rB1 and rB7 compared to CONTROLS, while percent predicted PEFR was significantly lower only in SUBJECTS and not in EMPHYSEMA, compared to CONTROLS. Fourthly, EMPHYSEMA in our study included variable levels of low attenuation areas assessed by modified Goddard scoring on chest CT despite all to be at the stage I level of chronic obstructive pulmonary disease (COPD) under the GOLD guidelines [5] (FEV1/FVC ratio in EMPHYSEMA in our study ranged from 58.4% to 69.9%).

### Strengths and limitations of this study

Since the slice thickness of our retrospectively acquired data ranged from 2 to 10 mm, this thickness might likely confound any measure of the intra-parenchymal airways size since very few are truly straight in the Z direction for such a distance. Those ranges of the slice thickness among subjects partially might explain why ratio of airway size to lung volume at rB7 between SUBJECTS and CONTROLS did not show a significantly difference. Because of the same reason, we could not directly measure lung volume. Nevertheless, the trachea was stable even in measuring 2–10 mm slice thicknesses. Thus, dysanaptic lung development is evident in SUBJECTS.

An abnormally low FEV1/FVC ratio is universally accepted as indicative of obstructive lung disease. Before recent revision, defining severity of stage I COPD has been set based solely on an FEV1/FVC less than 70% and FEV1 above 80% predicted irrespective of age [10]. However, later studies reported these criteria do not always work correctly [11]. The 2005ATS/ERS task force report defined obstructive abnormalities using the lower limit of normal (LLN), less than the 5th percentile predicted, of FEV1/FVC as the definition of obstruction. Although predictive equations for LLN of FEV1/FVC were variable [6] that is the estimation of the LLN from the different predicted normal equations gave variable results, SUBJECTS in our study were judged as within normal when we applied some of those criteria. This might suggest a physiological explanation behind the clinical significance of using LLN as the definition of obstruction. Nevertheless, the GOLD criteria still have been utilized widely in daily clinical practice including evaluation of pulmonary function test. The strength of our study lays in that the simple spirometry criteria which we set helps to explain the principle concept of dysanapsis as the normal variation of pulmonary mechanics. Due to its disproportionate growth between airway size to lung parenchyma, dysanapsis may lead to overdiagnosis of obstructive pulmonary disease in the situation where the GOLD criteria is used. In other words, we can attribute it as one of the possible causes of spirometry defined airflow limitation within the normal variation for healthy people. Awareness of these concept would help to interpret spirometry findings more accurately and may help to attenuate the risk of overdiagnosis in daily clinical practice, such as at health check up and/or preoperative screening processes.

We would like to acknowledge several limitations of our study. Our small sample size might have an effect on the study result. In addition, we could not assess the reversibility in airflow limitation, a recommended criterion for defining COPD [6]. Nevertheless, it is unlikely that SUBJECTS show the reversibility since none has any respiratory symptoms since birth and all are confirmed with normal lung field by chest CT. Furthermore, considering medical ethics of burdening invasion, it is beyond our scope to use the bronchodilator as part of the screening process. Furthermore, since we compared airway size at inhalation and the degree of airflow limitation at forced expiration, contribution of tracheal collapsibility was not accounted for. Small difference between the ratio of X-Ai to VC and the ratio of X-Ai to FVC may partially explain those contributions.

### Interpretation of findings in relation to previously published work

It is important to note the pathophysiology of SUBJECTS. Maximum airflow (Vmax) reflects the mechanical properties of lungs and airways [12,13]. It is determined by the static lung recoil pressure (Pst (l)) at that volume and the upstream resistance (Rus) between the alveoli and the equal pressure points (choke point) where intrabronchial pressure equals pleural pressure [14]. The Pst (l) depends on the absolute lung volume [15]. The larger FVC (≧100% of predicted FVC) influences the larger Pst (l) and the smaller airway area guides the higher Rus at the beginning of expiration. These would induce the reduction of maximum flow, and subsequently, a longer time to complete exhalation would be needed to produce FVC. Nevertheless, despite taking longer, they will still be able to produce more than 100% of predicted FEV1 as well as FVC since their driving pressure: Pst (l) is super normal. A relatively smaller X-Ai could lead to a reduction of expiratory maximum flow and to a decreased ratio of FEV1 to FVC, because the emptying rate of the lungs amortised over one second will be decreased due to the reduction of PEFR. Indeed, in healthy subjects PEFR predominantly reflects the caliber of large airways [16] and shows the significant positive correlation between tracheal size and PEFR [1,14,17]. This could explain the significant reduction of %PEFR in SUBJECTS but not in EMPHYSEMA compared to CONTROLS in our study results. Although we did not evaluate the expiratory time in our study, a previous study showed a longer expiratory time in the subjects who were suggested as dysanapsis [18], which support our idea.

As for origin of pulmonaly dysanapsis, its congenital basis as one of possibilities has been reported. Chen et al. [19], using segregation analysis of Vmax50 (the maximal expiratory flow rate at 50% of total volume) /FVC as a surrogate marker of dysanapsis, suggested that dysanaptic growth of the airways to parenchyma is under major gene control. Silverman et al. [20] showed significant linkage of FEV1/FVC to chromosome 2q could reflect one or more genes influencing the development of airflow obstruction or dysanapsis. Recently, Klimentidis et al. [21] found that the heritability for FEV1/FVC was considerably higher than the one for either FEV1 or FVC and demonstrated that genetic factors account for a sizable proportion of inter-individual differences in pulmonary function.

### Implications for future research, policy and practice

Dysanapsis could have a role in the pathogenesis of COPD [1] and asthma [22,23]. Observational study later showed that sons of COPD patients had a lower ratio of airway size to lung volume [24,25]. Thus, larger lungs are not necessarily advantageous unless the airway proportionally grows. Dysanapsis could be one of the risk for development of COPD, in addition to the generally accepted airflow limitation risks for COPD related with alternation of airway diameter secondary to airway remodeling [26,27]. Enrolled EMPHYSEMA in our study had a significant smaller ratio of airway size to lung volume compared to CONTROL when they showed variable levels of LAA on their chest CT. Actually, the severity of emphysematous lesion does not necessary reflect the airflow limitation defined by spirometry [28]. Dysanapsis could partially explain airflow limitation in our EMPHYSEMA patients, in addition to the elastic recoil destruction of the small conducting airways. In addition, annual decline of FEV1 is accelerated earlier than that of FVC [29]. The age-related effects on the lower FEV1/FVC is associated with an increase in tracheal cross sectional area [30-32], a decrease in airway radial distensibility [32,33] and elastic recoil [14,34-36]. However, age-related effects were less likely to have an effect on our study results because the study groups were adjusted for age.

## Conclusions

Our current study results suggest that spirometry defined airflow limitation by GOLD criteria with more than 100% of predicted FEV1 and FVC may suggest pulmonary dysanapsis as the normal variation in asymptomatic healthy subjects. Additional epidemiologic and physiologic studies with a large sample size are warranted in order to corroborate our results.

## Abbreviations

FEV_1_: Forced expiratory volume in one second
%FEV_1_: Percent predicted forced expiratory volume in one second
FVC: Forced vital capacity
%FVC: Percent predicted forced vital capacity
MMF: Maximum mid-expiratory flow
X-Ai: Cross sectional airway luminal area
PEFR: Peak expiratory flow
%PEFR: Percent predicted peak expiratory flow
CT: Computed tomography
WA: Wall area
Tr: Trachea above aorticarch
rB1: Right apical segmental bronchus
rB7: Right mediobasal segmental bronchus
COPD: Chronic obstructive pulmonary disease
LLN: Lower limit of normal
ATS: American Thoracic Society
ERS: European Respiratory Society

## References

[1] Green M, Mead J, Turner JM (1974). Variability of maximum expiratory flow-volume curves. J Appl Physiol.

[2] Mead J (1980). Dysanapsis in normal lungs assessed by the relationship between maximal flow, static recoil, and vital capacity. Am Rev Respir Dis.

[3] Hoffstein V (1986). Relationship between lung volume, maximal expiratory flow, forced expiratory volume in one second, and tracheal area in normal men and women. Am Rev Respir Dis.

[4] Tager IB, Weiss ST, Munoz A, Welty C, Speizer FE (1986). Determinants of response to eucapneic hyperventilation with cold air in a population-based study. Am Rev Respir Dis.

[5] Global Strategy for the Diagnosis, Management, and Prevention of Chronic Obstructive Pulmonary Disease. Global Initiative for Chronic Obstructive Lung Disease. 2011.

[6] Pellegrino R, Viegi G, Brusasco V, Crapo RO, Burgos F, Casaburi R (2005). Interpretative strategies for lung function tests. Eur Respir J.

[7] Diseases JSoC. Standards of pulmonary function tests for Japanese. 1993;31:appendix.

[8] San Jose Estepar RWG, Silverman EK, Reilly JJ, Kikinis R, Westin CF (2008). Airway inspector: an open source application for lung morphometry.

[9] Goddard PR, Nicholson EM, Laszlo G, Watt I (1982). Computed tomography in pulmonary emphysema. Clin Radiol.

[10] Global Initiative for Chronic Obstructive Lung Disease. Global strategy for the diagnosis, management and prevention of chronic obstructive lung disease. Updated 2010. Available at: http://www.goldcopd.com. Accessed December 2014.

[11] Celli BR, Halbert RJ, Isonaka S, Schau B (2003). Population impact of different definitions of airway obstruction. Eur Respir J.

[12] Hyatt RE, Flath RE (1966). Relationship of air flow to pressure during maximal respiratory effort in man. J Appl Physiol.

[13] Hyatt R. Forced expiration. In: Fishman AP, editor. Handboole of physiology. Oxford University Press; 1986.

[14] Mead J, Turner JM, Macklem PT, Little JB (1967). Significance of the relationship between lung recoil and maximum expiratory flow. J Appl Physiol.

[15] Fry DL, Hyatt RE (1960). Pulmonary mechanics. A unified analysis of the relationship between pressure, volume and gasflow in the lungs of normal and diseased human subjects. Am J Med.

[16] Hughes JMB PN (1999). Lung Function Tests: Physiological Principles and Clinical Applications.

[17] Osmanliev D, Bowley N, Hunter DM, Pride NB (1982). Relation between tracheal size and forced expiratory volume in one second in young men. Am Rev Respir Dis.

[18] Barisione G, Crimi E, Bartolini S, Saporiti R, Copello F, Pellegrino R (2009). How to interpret reduced forced expiratory volume in 1 s (FEV1)/vital capacity ratio with normal FEV1. Eur Respir J.

[19] Chen Y, Dosman JA, Rennie DC, Lockinger LA (1999). Major genetic effects on airway-parenchymal dysanapsis of the lung: the Humboldt family study. Genet Epidemiol.

[20] Silverman EK, Palmer LJ, Mosley JD, Barth M, Senter JM, Brown A (2002). Genomewide linkage analysis of quantitative spirometric phenotypes in severe early-onset chronic obstructive pulmonary disease. Am J Hum Genet.

[21] Klimentidis YC, Vazquez AI, de Los CG, Allison DB, Dransfield MT, Thannickal VJ (2013). Heritability of pulmonary function estimated from pedigree and whole-genome markers. Front Genet.

[22] Munakata M, Ohe M, Homma Y, Kawakami Y (1997). Pulmonary dysanapsis, methacholine airway responsiveness and sensitization to airborne antigen. Respirology.

[23] Parker AL, McCool FD (2002). Pulmonary function characteristics in patients with different patterns of methacholine airway hyperresponsiveness. Chest.

[24] Nishimura M, Kusaka T, Kobayashi S, Yamamoto M, Akiyama Y, Kawakami Y (1991). [Analysis of dysanapsis in healthy twins and sons of patients with chronic obstructive lung disease]. Nihon Kyobu Shikkan Gakkai Zasshi.

[25] Kawakami Y, Irie T, Kishi F, Asanuma Y, Shida A, Yoshikawa T (1981). Familial aggregation of abnormal ventilatory control and pulmonary function in chronic obstructive pulmonary disease. Eur J Respir Dis.

[26] Hogg JC, Macklem PT, Thurlbeck WM (1968). Site and nature of airway obstruction in chronic obstructive lung disease. N Engl J Med.

[27] Yanai M, Sekizawa K, Ohrui T, Sasaki H, Takishima T (1992). Site of airway obstruction in pulmonary disease: direct measurement of intrabronchial pressure. J Appl Physiol.

[28] Bhatt SP, Sieren JC, Dransfield MT, Washko GR, Newell JD, Stinson DS (2014). Comparison of spirometric thresholds in diagnosing smoking-related airflow obstruction. Thorax.

[29] Hankinson JL, Odencrantz JR, Fedan KB (1999). Spirometric reference values from a sample of the general U.S. population. Am J Respir Crit Care Med.

[30] Collins DV, Cutillo AG, Armstrong JD, Crapo RO, Kanner RE, Tocino I (1986). Large airway size, lung size, and maximal expiratory flow in healthy nonsmokers. Am Rev Respir Dis.

[31] Gibellino F, Osmanliev DP, Watson A, Pride NB (1985). Increase in tracheal size with age. Implications Maximal Expiratory Am Rev Respir Dis.

[32] Breatnach E, Abbott GC, Fraser RG (1984). Dimensions of the normal human trachea. AJR Am J Roentgenol.

[33] Wilson AG, Massarella GR, Pride NB (1974). Elastic properties of airways in human lungs post mortem. Am Rev Respir Dis.

[34] Turner JM, Mead J, Wohl ME (1968). Elasticity of human lungs in relation to age. J Appl Physiol.

[35] Gibson GJ, Pride NB, O’Cain C, Quagliato R (1976). Sex and age differences in pulmonary mechanics in normal nonsmoking subjects. J Appl Physiol.

[36] Knudson RJ, Clark DF, Kennedy TC, Knudson DE (1977). Effect of aging alone on mechanical properties of the normal adult human lung. J Appl Physiol Respir Environ Exerc Physiol.

